# The kynurenine pathway: an immunometabolic bridge linking systemic inflammation to neuroaxonal vulnerability in multiple sclerosis

**DOI:** 10.3389/fncel.2026.1831349

**Published:** 2026-05-25

**Authors:** José Luis Maldonado-García, Lissette Haydee García-Mena, Eduardo Alberto Ferat-Peláez, Gilberto Pérez-Sánchez, Enrique Becerril-Villanueva, Argelia Esperanza Rojas-Mayorquín, Manuel Iván Girón-Pérez, Daniel Ortuño-Sahagún, Lenin Pavón

**Affiliations:** 1 Departamento de Bioquímica, Facultad de Medicina, Universidad Nacional Autónoma de México, Coyoacán, Ciudad de México, Mexico; 2 Coordinación Académica, Licenciatura en Medicina, Escuela de Ciencias de la Salud, Universidad del Valle de México Campus Coyoacán, Coyoacán, Ciudad de México, Mexico; 3 Departamento de Salud Digital, Facultad de Medicina, Universidad Nacional Autónoma de México, Coyoacán, Ciudad de México, Mexico; 4 Facultad Mexicana de Medicina, Universidad La Salle, Tlalpan, Ciudad de México, Mexico; 5 Laboratorio de Psicoinmunología, Instituto Nacional de Psiquiatría Ramón de la Fuente Muñiz, Mexico City, Mexico; 6 Departamento Materno Infantil, Centro Universitario de Tlajomulco, Universidad de Guadalajara, Tlajomulco, Jalisco, Mexico; 7 Laboratorio Nacional LANIIA-NAYARIT, Universidad Autónoma de Nayarit, Tepic, Nayarit, Mexico; 8 Laboratorio de Neuroinmunobiología Molecular, Instituto de Neurociencias Traslacionales, Centro Universitario de Ciencias de la Salud, Universidad de Guadalajara, Guadalajara, Jalisco, Mexico

**Keywords:** inflammation, kynurenic acid, kynurenines pathway, multiple sclerosis, pro-inflammatory cytokines, quinolinic acid

## Abstract

Multiple sclerosis (MS) extends beyond focal autoimmune demyelination, with progressive neurodegeneration and cognitive impairment arising from mechanisms not fully explained by inflammatory lesions alone. We propose the kynurenine pathway (KP) as a unifying immunometabolic interface that translates peripheral inflammation into central neuroaxonal vulnerability. Cytokine-driven induction of indoleamine 2,3-dioxygenase accelerates systemic tryptophan catabolism, increasing circulating kynurenine that crosses the blood–brain barrier via L-type amino acid transporter 1 (LAT1). Within the CNS, microglial kynurenine 3-monooxygenase generates the excitotoxic and pro-oxidant metabolites quinolinic acid and 3-hydroxykynurenine, while astrocytic kynurenine aminotransferases produce the neuroprotective antagonist kynurenic acid. This enzymatic dichotomy creates a dynamic amplification loop that intensifies excitotoxicity, oxidative stress, oligodendrocyte injury, and axonal loss. Clinical evidence shows that kynurenine metabolite ratios correlate with plasma neurofilament light chain, cognitive deficits, obesity-related inflammation, and phenotype-specific exercise responsiveness, underscoring KP plasticity as a potential determinant of disease trajectory. By reframing MS through this immunometabolic lens, the KP emerges as both a mechanistic bridge between inflammation and neurodegeneration and a tractable biomarker system with therapeutic potential.

## 1 Introduction

Multiple sclerosis (MS) is a chronic immune-mediated disorder of the central nervous system characterized by inflammatory demyelination, axonal injury, and progressive neurological disability (Filippi et al., 2018). Although focal immune cell infiltration and autoreactive lymphocyte activity remain central to disease pathogenesis, increasing evidence indicates that chronic neurodegeneration and cognitive impairment cannot be fully explained by inflammatory lesions alone (Filippi et al., 2018). Cognitive dysfunction affects approximately 40–65% of individuals with MS, frequently involving information-processing speed, executive function, and memory, and may occur even in the early stages of the disease (Benedict et al., 2020; Amato et al., 2010).

**Figure 1:**
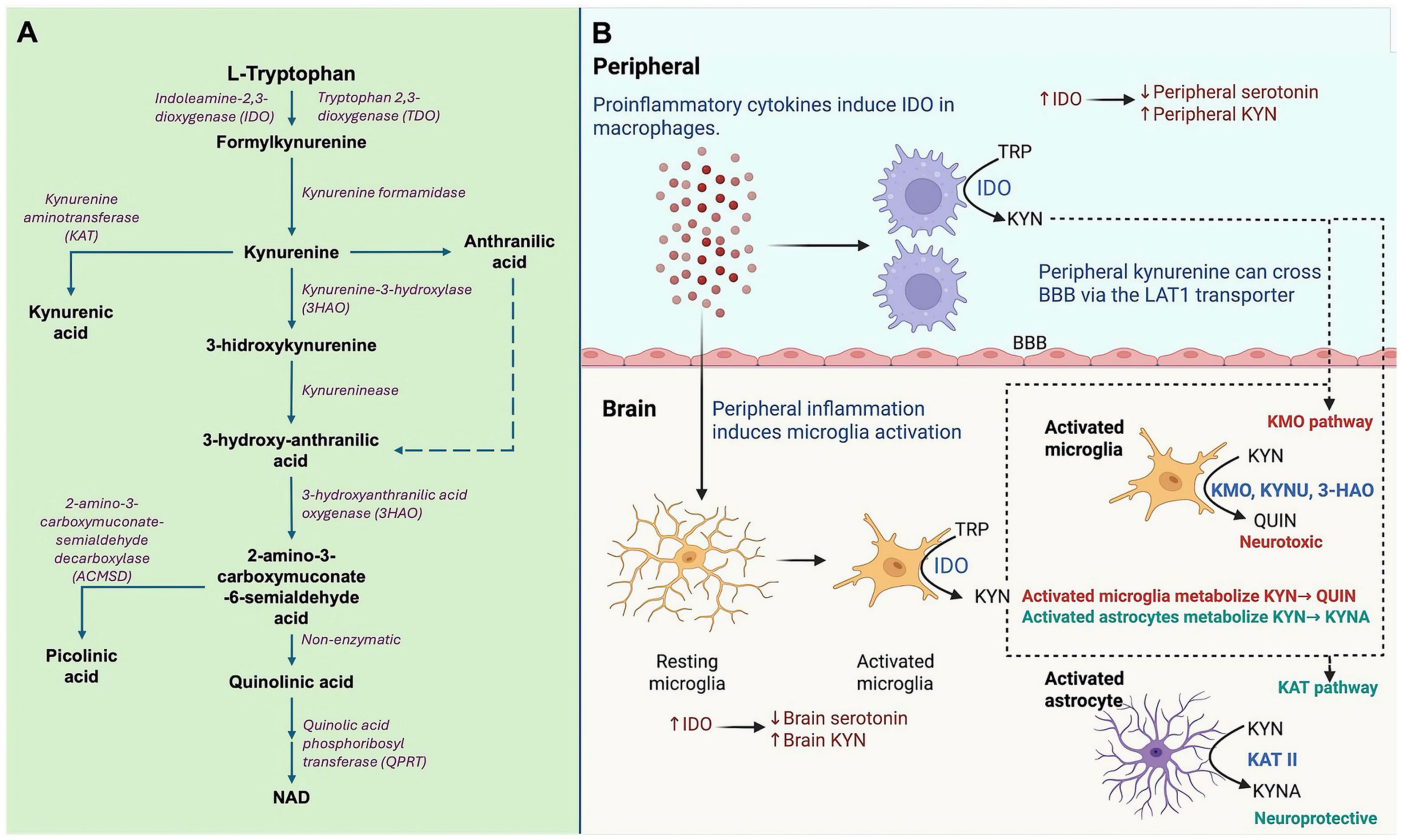
The kynurenine pathway as a peripheral–central immunometabolic interface in multiple sclerosis. **(A)** Overview of the kynurenine pathway (KP), depicting the conversion of L-tryptophan into kynurenine via indoleamine 2,3-dioxygenase (IDO) and tryptophan 2,3-dioxygenase (TDO), and its subsequent metabolism into neurotoxic (e.g., 3-hydroxykynurenine [3-HK], quinolinic acid [QA]) and neuroprotective (kynurenic acid [KYNA]) metabolites. **(B)** Peripheral–central coupling of KP metabolism. Inflammatory cytokines induce IDO activity, increasing circulating kynurenine and reducing serotonin availability. Kynurenine crosses the blood–brain barrier via L-type amino acid transporter 1 (LAT1) and undergoes cell-specific metabolism: microglial KMO activity promotes neurotoxic metabolites, whereas astrocytic KAT activity favors kynurenic acid production. This balance links systemic inflammation with excitotoxicity, oxidative stress, and neuroaxonal vulnerability in multiple sclerosis.

Beyond classical immune mechanisms, metabolic alterations have gained attention as modulators of neuroinflammatory vulnerability. In this context, the kynurenine pathway (KP), the principal route of tryptophan degradation, has emerged as a relevant immunometabolic interface between systemic inflammation and central nervous system function. The pathway initiates with the conversion of L-tryptophan into N-formylkynurenine through the enzymatic activity of indoleamine 2,3-dioxygenase 1 (IDO1) in extrahepatic tissues and tryptophan 2,3-dioxygenase (TDO) in the liver. N-formylkynurenine is rapidly converted into kynurenine, the central metabolite of the pathway, which serves as a branching point toward both neurotoxic and neuroprotective downstream products. Under inflammatory conditions, pro-inflammatory cytokines such as interferon-γ and tumor necrosis factor-α further induce IDO1, increasing peripheral tryptophan catabolism and elevating circulating kynurenine levels (Figure 1A) (Stone et al., 2022; Campbell et al., 2014; Wang et al., 2025).

Kynurenine readily crosses the blood–brain barrier via the L-type amino acid transporter 1 (LAT1), where it undergoes differential metabolism in microglia and astrocytes. Microglial kynurenine 3-monooxygenase (KMO) promotes the production of neurotoxic metabolites such as quinolinic acid (QA) and 3-hydroxykynurenine (3-HK), whereas astrocytic kynurenine aminotransferases favor the generation of kynurenic acid (KYNA), a metabolite with neuroprotective properties (Stone et al., 2022; Campbell et al., 2014; Pavón et al., 2024; Maldonado-García et al., 2024).

In MS, dysregulation of this metabolic balance has been associated with markers of neuroaxonal injury. Clinical studies have reported that serum kynurenine metabolite profiles correlate with plasma neurofilament light chain (pNfL), a validated biomarker of axonal damage, and differ according to body mass index and inflammatory status (Kupjetz et al., 2023). Moreover, acute physical exercise has been shown to modulate kynurenine flux and reduce pNfL levels in patients with relapsing–remitting MS, suggesting that KP dynamics may reflect disease-related metabolic plasticity (Joisten et al., 2021). From this perspective, the KP may represent a significant immunometabolic interface through which systemic immune activation could affect neuroaxonal injury and cognitive vulnerability in MS.

## 2 The kynurenine pathway as a peripheral–central immunometabolic interface

Chronic immune activation is a defining feature of MS, characterized by sustained production of pro-inflammatory cytokines such as interferon-γ (IFN-γ), tumor necrosis factor-α (TNF-α), and interleukin-1β (IL-1β). These mediators orchestrate immune cell trafficking, lesion formation, and induce profound metabolic shifts (Stone et al., 2022; Venkatesan et al., 2020; Zádor et al., 2021). Among these responses, activation of the KP represents a major immunometabolic adaptation to inflammation. IFN-γ strongly induces IDO1, the rate-limiting enzyme in extrahepatic tryptophan degradation, resulting in increased conversion of tryptophan to kynurenine and elevation of the kynurenine-to-tryptophan ratio (KTR) in peripheral blood (Stone et al., 2022; Campbell et al., 2014; Zádor et al., 2021).

Kynurenine is the principal KP metabolite that efficiently crosses the blood–brain barrier via LAT1, thereby linking peripheral immune activation with central nervous system metabolism (Figure 1B) (Skorobogatov et al., 2021). This positions kynurenine as a biochemical interface between systemic inflammatory signals and central metabolic processes. Once in the brain parenchyma, kynurenine undergoes cell-type–specific metabolism. Microglia preferentially express KMO, directing kynurenine toward the production of 3-HK and QA, metabolites associated with oxidative stress and N-methyl-D-aspartate (NMDA) receptor activation. In contrast, astrocytes predominantly express kynurenine aminotransferases (KATs), facilitating the synthesis of KYNA, an NMDA receptor antagonist with neuroprotective properties (Campbell et al., 2014; Skorobogatov et al., 2021; Lugo-Huitrón et al., 2013).

This differential enzymatic distribution establishes a dynamic balance between the neurotoxic and neuroprotective branches of the pathway. In inflammatory contexts, increased IDO1 activity and kynurenine availability may favor microglial KMO-dependent metabolism, potentially shifting the QA/KYNA equilibrium toward excitotoxic and pro-oxidative conditions. Experimental and clinical observations in MS suggest that changes in kynurenine metabolite profiles may have functional relevance rather than being secondary by-products of inflammation. Serum KP signatures have been associated with markers of axonal injury, including pNfL, and appear to vary according to inflammatory and metabolic status (Kupjetz et al., 2023).

Importantly, this peripheral–central coupling does not imply that KP dysregulation acts independently of classical immune mechanisms. Rather, it may operate as a metabolic intermediary through which systemic inflammatory signals modulate central vulnerability. By influencing excitotoxic tone, redox balance, and glial activation states, kynurenine flux may contribute to the amplification of neuroaxonal stress in MS. Within this framework, the KP can be conceptualized as an interface integrating immune activation with metabolic regulation in the central nervous system, without assuming direct causality but acknowledging its potential modulatory role.

## 3 From metabolic flux to neuroaxonal injury and cognitive vulnerability

Alterations in KP metabolism may influence central nervous system integrity through mechanisms that converge on excitotoxicity, oxidative stress, and glial dysfunction. Quinolinic acid (QA), a downstream product of the microglial kynurenine KMO branch, acts as an NMDA receptor agonist, promoting glutamatergic overstimulation and intracellular calcium influx. Sustained NMDA activation has been associated with mitochondrial dysfunction, increased production of reactive oxygen and nitrogen species, and disruption of cellular bioenergetics (Campbell et al., 2014; Stone and Williams, 2023).

In parallel, 3-HK exerts pro-oxidative effects and may further amplify redox imbalance within activated microglia and neural cells. This biochemical environment is particularly relevant in MS, where chronic microglial activation and persistent low-grade inflammation characterize both active lesions and smoldering pathology in progressive stages (Campbell et al., 2014; Skorobogatov et al., 2021; Mor et al., 2021).

Beyond neuronal excitotoxicity, oligodendrocytes are especially vulnerable to oxidative stress and mitochondrial impairment (Emamnejad et al., 2024). Although direct causal relationships remain under investigation, QA-induced excitotoxicity and redox dysregulation may plausibly exacerbate demyelination and impair remyelination capacity (Emamnejad et al., 2024). In this context, KP dysregulation could contribute to axonal stress not only through neuronal mechanisms but also by influencing glial–axonal metabolic coupling. Clinical observations support this mechanistic framework. Serum kynurenine metabolite ratios, particularly those reflecting neurotoxic predominance, have been associated with pNfL, a validated biomarker of axonal injury in MS (Kupjetz et al., 2023). Because axonal loss is a major determinant of long-term disability and cognitive decline in MS, these associations suggest that peripheral KP signatures may parallel central neuroaxonal stress. Cognitive impairment in MS – often involving processing speed, executive function, and memory – has been linked to structural and functional disruption of hippocampal and fronto-subcortical networks (Benedict et al., 2020; Amato et al., 2010).

Although direct longitudinal data remain limited, the convergence of inflammatory activation, altered kynurenine metabolite balance, and biomarkers of axonal damage raises the possibility that KP dynamics may participate in modulating cognitive vulnerability in MS. Importantly, this perspective does not position the KP as an isolated driver of pathology but rather as a metabolic intermediary that may amplify existing inflammatory and neurodegenerative processes.

## 4 Metabolic plasticity across MS phenotypes

The clinical heterogeneity of MS suggests that inflammatory and neurodegenerative processes do not operate uniformly across disease stages. Relapsing–remitting MS (RRMS) is characterized by episodic inflammatory activity, whereas secondary progressive MS (SPMS) involves more sustained neurodegeneration with attenuated overt inflammatory relapses (Filippi et al., 2018). In this context, differences in KP dynamics may reflect underlying variations in immunometabolic adaptability.

Recent evidence indicates that acute physical exercise modulates KP metabolism in patients with RRMS. High-intensity interval training has been associated with reductions in pNfL and shifts in kynurenine flux toward increased production of kynurenic acid (KYNA), suggesting a transient enhancement of the neuroprotective branch of the pathway (Joisten et al., 2021).

In contrast, this metabolic response appears diminished or absent in SPMS, where exercise-induced changes in the kynurenine-to-tryptophan ratio (KTR) and downstream metabolites are less pronounced. These observations raise the possibility that progressive stages of MS may involve reduced metabolic flexibility, potentially limiting the capacity to counterbalance neurotoxic metabolite accumulation (Joisten et al., 2021).

Body mass index (BMI) and systemic metabolic status may further modulate this balance. Elevated BMI in MS has been associated with increased KTR and greater accumulation of downstream neurotoxic metabolites, including quinolinic acid (QA) and 3-hydroxykynurenine (3-HK). These findings suggest that chronic low-grade inflammation linked to adiposity may amplify peripheral kynurenine flux and influence central vulnerability (Kupjetz et al., 2023).

Taken together, these observations support the concept that KP regulation in MS is not static but dynamically responsive to inflammatory and metabolic context. Rather than serving solely as a biochemical marker of immune activation, kynurenine flux may reflect a broader capacity for metabolic adaptation. A diminished ability to redirect kynurenine metabolism toward the KYNA-producing branch could, in theory, favor sustained exposure to neurotoxic metabolites and contribute to progressive neuroaxonal stress.

Importantly, these interpretations remain preliminary. Longitudinal studies integrating KP metabolite profiling, imaging biomarkers, and clinical outcomes are required to determine whether alterations in metabolic adaptability precede or follow neurodegenerative progression. Nevertheless, the emerging phenotype-dependent differences in KP responsiveness provide a framework for considering MS as a disorder in which immunometabolic plasticity may influence disease trajectory.

## 5 Clinical and translational implications

Emerging evidence identifies the KP as a peripheral–central immunometabolic interface in MS, with important translational implications. One of the most accessible clinical indices of KP activation is the KTR, commonly used as a surrogate marker for IDO1 activity. Elevated KTR has been associated with systemic inflammatory tone and metabolic status, and in MS, changes in kynurenine metabolite profiles have been linked to pNfL, a validated biomarker of axonal injury (Stone et al., 2022; Kupjetz et al., 2023; Lugo-Huitrón et al., 2013; Kupjetz et al., 2025). These observations suggest that peripheral KP metrics may complement existing neuroaxonal biomarkers by providing a dynamic measure of immunometabolic activity rather than structural damage alone. Integrating KP profiling with pNfL measurements may therefore improve risk stratification and disease monitoring, although prospective validation is still needed before clinical implementation.

The therapeutic landscape of MS has changed substantially over the past decade, expanding from platform therapies such as interferon beta-1a (IFNβ-1a) and glatiramer acetate to a broader range of disease-modifying therapies (DMTs) with distinct mechanisms of action (Filippi et al., 2018). Current strategies include oral agents such as fumarates and sphingosine-1-phosphate receptor modulators, as well as high-efficacy monoclonal antibodies targeting immune cell trafficking or depletion, including anti-CD20 therapies (e.g., ocrelizumab and ofatumumab) and natalizumab. While these treatments effectively reduce inflammatory activity, relapse rates, and disability progression, their impact on immunometabolic pathways, including the KP, remains incompletely characterized, particularly for contemporary high-efficacy therapies (Filippi et al., 2018; Rashid et al., 2025; Susin-Calle et al., 2025).

Within this evolving therapeutic landscape, immunomodulatory therapies may influence KP dynamics, although the nature and extent of this interaction appear heterogeneous. The most consistent human evidence comes from IFNβ-1a, which has been shown to increase KTR through stimulation of IDO activity, reflecting engagement with inflammatory signaling pathways. Conversely, the development of neutralizing antibodies against IFNβ-1a has been associated with reduced KTR, suggesting that kynurenine metrics may capture aspects of therapeutic responsiveness (Durastanti et al., 2011).

Recent metabolomic studies suggest that anti-CD20 therapies such as ocrelizumab may also influence tryptophan metabolism, with treated patients showing partial normalization of kynurenine-related metabolic profiles compared to baseline or untreated states (Siavoshi et al., 2024; Ricci et al., 2025). Although these findings are based on peripheral metabolomic analyses and remain preliminary, they provide emerging human evidence that contemporary high-efficacy therapies may modulate immunometabolic pathways beyond their established immunological effects.

Additional clinical data support a predominantly indirect interaction between current DMTs and the KP. Fingolimod has been associated with altered IDO expression and pro-inflammatory cytokine profiles in treated patients (Tauil et al., 2020), while therapies such as natalizumab and dimethyl fumarate primarily act by modulating immune cell trafficking and inflammatory signaling networks that are mechanistically upstream of KP activation (Mellerård et al., 2010; Holm Hansen et al., 2019). Therefore, their influence on the KP is likely mediated through changes in systemic immune activation rather than direct enzymatic regulation.

Supporting this concept, recent clinical observations have reported reduced peripheral serotonin levels in patients with MS receiving IFNβ-1a, a finding consistent with increased diversion of tryptophan metabolism away from serotonin synthesis toward the KP (Pérez-Sánchez et al., 2026). Although serotonin is not a direct metabolite of the KP, these findings provide indirect human evidence of treatment-associated shifts in tryptophan metabolism, reinforcing the concept of the KP as a responsive immunometabolic interface.

Lifestyle interventions further underscore the modulatory potential of this pathway. Structured physical exercise has been associated with shifts in kynurenine metabolism toward increased kynurenic acid (KYNA) production and reductions in pNfL in relapsing–remitting MS, supporting the concept that KP dynamics respond to systemic metabolic signals (Kupjetz et al., 2023; Joisten et al., 2021; Javelle et al., 2025).

Collectively, these findings suggest that the kynurenine pathway (KP) may function not only as a mechanistic intermediary but also as a dynamic biomarker system reflecting inflammatory activity, metabolic context, and potentially therapeutic engagement. Taking together, this body of evidence supports the concept that the KP operates as an immunometabolic interface linking systemic inflammatory signals with neuroaxonal stress. However, current data are largely cross-sectional or derived from short-term interventions. Standardization of metabolite measurements, clarification of central–peripheral concordance, and longitudinal validation will be essential before routine clinical integration can be considered. The principal mechanisms underlying this framework and their potential clinical implications are summarized in Table 1.

**Table 1:** The kynurenine pathway as an immunometabolic interface linking systemic inflammation with neuroaxonal vulnerability in multiple sclerosis.

Concept	Mechanistic basis	Clinical and translational relevance
Systemic activation of the kynurenine pathway	Pro-inflammatory cytokines (IFN-γ, TNF-α, IL-1β) induce indoleamine-2,3-dioxygenase-1 (IDO1), increasing tryptophan degradation and elevating the kynurenine-to-tryptophan ratio (KTR). Kynurenine crosses the blood–brain barrier through the LAT1 transporter.	Links peripheral immune activation with central nervous system (CNS) metabolic responses, positioning the KP as a peripheral–central immunometabolic interface.
Microglial neurotoxic branch	Microglia preferentially express kynurenine-3-monooxygenase (KMO), converting kynurenine into neurotoxic metabolites such as quinolinic acid (QA) and 3-hydroxykynurenine (3-HK).	QA activates NMDA receptors, promoting excitotoxicity, oxidative stress, and mitochondrial dysfunction, processes associated with axonal injury and neurodegeneration.
Astrocytic neuroprotective branch	Astrocytes express kynurenine aminotransferases (KATs), directing kynurenine metabolism toward kynurenic acid (KYNA).	KYNA acts as an NMDA receptor antagonist and exerts neuroprotective effects, potentially counterbalancing excitotoxic and oxidative mechanisms.
Biomarker associations	Alterations in KP metabolite balance, particularly increased neurotoxic metabolites or elevated KTR.	Associated with plasma neurofilament light chain (pNfL), a validated biomarker of axonal injury, suggesting that peripheral KP signatures may reflect central neuroaxonal stress and cognitive vulnerability.
Metabolic plasticity and exercise	Physical exercise can modulate KP flux by enhancing peripheral metabolism of kynurenine toward KYNA-producing pathways.	In relapsing–remitting MS (RRMS), exercise has been associated with reduced pNfL levels and a shift toward the neuroprotective branch of the pathway; this adaptive response appears diminished in progressive MS phenotypes.
Influence of metabolic status (BMI)	Overweight and obesity are associated with chronic low-grade inflammation and increased IDO activity, promoting higher KTR and accumulation of downstream neurotoxic metabolites.	Suggests that systemic metabolic factors may amplify kynurenine flux and influence neuroaxonal vulnerability in MS.
Pharmacological modulation	Immunomodulatory therapies such as interferon β-1a can stimulate IDO activity and modify KP dynamics.	Changes in KTR during treatment may reflect biological engagement with immune pathways and could potentially serve as indicators of therapeutic responsiveness.

### 5.1 Systemic implications

Beyond its relevance within the CNS, KP dysregulation reflects systemic immunometabolic states that may indirectly shape central vulnerability. Although the present perspective focuses on MS, KP dysregulation is not restricted to the central nervous system. In chronic inflammatory and metabolic conditions, including obesity and chronic kidney disease, altered kynurenine flux and accumulation of downstream metabolites have been reported (Kozieł and Urbanska, 2023; El Chamieh et al., 2025). These systemic alterations may influence circulating kynurenine availability and thus modulate central exposure (Stone et al., 2022). While direct implications for MS remain to be fully elucidated, these findings underscore the importance of considering comorbid metabolic and inflammatory states when interpreting KP profiles in neurological disease.

## 6 Discussion

This perspective proposes that the KP may function as a relevant immunometabolic interface linking peripheral inflammatory activation with central neuroaxonal vulnerability in MS. Rather than viewing KP dysregulation as an isolated driver of pathology, the evidence reviewed here supports a model in which peripheral cytokine-mediated induction of tryptophan degradation increases circulating kynurenine availability, thereby influencing central metabolite balance through cell-type–specific enzymatic pathways. Within the central nervous system, microglial KMO-dependent metabolism and astrocytic KAT activity determine the relative predominance of neurotoxic and neuroprotective branches, potentially affecting excitotoxic tone, redox homeostasis, and glial–axonal interactions.

This framework provides a complementary perspective to classical immune-centric models of MS. While autoreactive lymphocytes and lesion formation remain central to disease pathogenesis, metabolic intermediaries such as kynurenines may modulate the intensity and persistence of neuroaxonal stress. Associations between peripheral KP metabolite profiles and plasma pNfL, as well as phenotype-dependent differences in exercise responsiveness, suggest that kynurenine flux reflects dynamic aspects of disease biology rather than static inflammatory markers. These findings support the concept that MS progression involves not only immune activation but also altered metabolic adaptability.

Importantly, the current evidence is largely associative. Most clinical data are cross-sectional or derived from short-term interventions, and direct longitudinal links between KP dynamics and structural neurodegeneration are limited. Central–peripheral concordance of kynurenine metabolites requires further clarification, as does the temporal sequence between inflammatory activation, metabolic shifts, and axonal injury. Standardization of analytical platforms and harmonization of metabolite panels will be critical for reproducibility across cohorts.

Future research directions should prioritize integrated longitudinal studies combining KP metabolite profiling, advanced neuroimaging, fluid biomarkers such as pNfL, and detailed cognitive phenotyping. Experimental models that selectively modulate KMO or KAT activity may further clarify whether shifting the neurotoxic – neuroprotective balance alters disease trajectory. In parallel, the influence of systemic metabolic factors – including adiposity, exercise capacity, and comorbid inflammatory conditions – warrants systematic evaluation to determine whether modifying peripheral immunometabolic tone can influence central vulnerability.

## 7 Conclusions and future perspectives

In conclusion, the KP provides a biologically plausible and clinically relevant framework for understanding how peripheral immune signals may intersect with central neurodegenerative processes in MS. Serving as a critical immunometabolic interface through which peripheral cytokine-driven tryptophan degradation modulates central metabolite balance and increases excitotoxic and oxidative stress, leading to neuroaxonal injury. This framework is supported by consistent associations between KTR, pNfL, and cognitive impairment, as well as phenotype-dependent differences in metabolic plasticity observed in response to exercise and adiposity. Future research must address key gaps to advance the field.

Current evidence is largely cross-sectional or based on short-term interventions; longitudinal studies integrating serial kynurenine profiling, advanced neuroimaging, pNfL, and cognitive phenotyping are essential to establish temporal relationships and causality. Central–peripheral metabolite concordance, analytical standardization, and harmonization of panels require urgent resolution for reproducibility. Experimental models that selectively modulate KMO or KAT will clarify whether restoring the neurotoxic–neuroprotective balance alters disease progression. Systematic evaluation of systemic metabolic comorbidities, such as adiposity and inflammation, is also needed to define actionable strategies to enhance metabolic resilience. While definitive causal relationships remain to be established, recognizing KP dynamics as part of the broader immunometabolic landscape of MS may inform future biomarker development and therapeutic exploration.

## Data Availability

The original contributions presented in the study are included in the article/supplementary material, further inquiries can be directed to the corresponding authors.

## In memoriam

We dedicate this work to the memory of Dr. Francisco Raúl Chávez Sánchez, a pioneer of immunology at the Faculty of Medicine of the Universidad Nacional Autónoma de México. His vision and leadership were instrumental in advancing immunology within the institution and in shaping the careers of numerous physicians and scientists. Beyond his academic achievements, he was an exceptional mentor whose generosity and human quality left a lasting impact on those he guided. His legacy endures in the generations he inspired. JLM-G gratefully acknowledges the opportunity he provided at the beginning of his academic career.

## References

[ref1] Amato M. P. Portaccio E. Goretti B. Zipoli V. Hakiki B. Giannini M. . (2010). Cognitive impairment in early stages of multiple sclerosis. Neurol. Sci. 31, 211–214. doi: 10.1007/s10072-010-0376-4 20640466

[ref2] Benedict R. H. B. Amato M. P. DeLuca J. Geurts J. J. G. (2020). Cognitive impairment in multiple sclerosis: clinical management, MRI, and therapeutic avenues. Lancet Neurol. 19, 860–871. doi: 10.1016/S1474-4422(20)30277-5, 32949546 PMC10011205

[ref3] Campbell B. M. Charych E. Lee A. W. Möller T. (2014). Kynurenines in CNS disease: regulation by inflammatory cytokines. Front. Neurosci. 8:12. doi: 10.3389/fnins.2014.00012, 24567701 PMC3915289

[ref4] Durastanti V. Lugaresi A. Bramanti P. Amato M. Bellantonio P. De Luca G. . (2011). Neopterin production and tryptophan degradation during 24-months therapy with interferon beta-1a in multiple sclerosis patients. J. Transl. Med. 9:42. doi: 10.1186/1479-5876-9-42, 21501517 PMC3102623

[ref5] El Chamieh C. Liabeuf S. Larabi I. A. De Pinho N. A. Costes-Albrespic M. Frimat L. . (2025). Uncovering the link between kynurenic acid pathway and kidney failure. Kidney Int Rep 10, 1404–1414. doi: 10.1016/j.ekir.2025.02.024, 40485675 PMC12142646

[ref6] Emamnejad R. Pagnin M. Petratos S. (2024). The iron maiden: Oligodendroglial metabolic dysfunction in multiple sclerosis and mitochondrial signaling. Neurosci. Biobehav. Rev. 164:105788. doi: 10.1016/j.neubiorev.2024.105788, 38950685

[ref7] Filippi M. Bar-Or A. Piehl F. Preziosa P. Solari A. Vukusic S. . (2018). Multiple sclerosis. Nat. Rev. Dis. Primers 4:43. doi: 10.1038/s41572-018-0041-4 30410033

[ref8] Holm Hansen R. Højsgaard Chow H. Sellebjerg F. Rode von Essen M. (2019). Dimethyl fumarate therapy suppresses B cell responses and follicular helper T cells in relapsing-remitting multiple sclerosis. Mult. Scler. 25, 1289–1297. doi: 10.1177/1352458518790417, 30043661

[ref9] Javelle F. Bloch W. Koppe K. Krombholz S. Thevis M. Wanka L. . (2025). Physical exercise to rebalance kynurenine metabolism in borderline personality disorder - preliminary findings. Brain Behav Immun Health 47:101030. doi: 10.1016/j.bbih.2025.101030, 40585351 PMC12205827

[ref10] Joisten N. Rademacher A. Warnke C. Proschinger S. Schenk A. Walzik D. . (2021). Exercise diminishes plasma neurofilament light chain and reroutes the kynurenine pathway in multiple sclerosis. Neurol Neuroimmunol Neuroinflamm 8:982. doi: 10.1212/NXI.0000000000000982, 33782190 PMC8054957

[ref11] Kozieł K. Urbanska E. M. (2023). Kynurenine pathway in diabetes mellitus-novel pharmacological target? Cells 12:460. doi: 10.3390/cells12030460, 36766803 PMC9913876

[ref12] Kupjetz M. Patt N. Joisten N. Ueland P. M. McCann A. Gonzenbach R. . (2023). The serum kynurenine pathway metabolic profile is associated with overweight and obesity in multiple sclerosis. Mult. Scler. Relat. Disord. 72:104592. doi: 10.1016/j.msard.2023.104592, 36881945

[ref13] Kupjetz M. Wences Chirino T. Y. Joisten N. Zimmer P. (2025). Kynurenine pathway dysregulation as a mechanistic link between cognitive impairment and brain damage: implications for multiple sclerosis. Brain Res. 1853:149415. doi: 10.1016/j.brainres.2024.149415, 39710050

[ref14] Lugo-Huitrón R. Ugalde Muñiz P. Pineda B. Pedraza-Chaverrí J. Ríos C. Pérez-De La Cruz V. (2013). Quinolinic acid: an endogenous neurotoxin with multiple targets. Oxidative Med. Cell. Longev. 2013:104024. doi: 10.1155/2013/104024, 24089628 PMC3780648

[ref15] Maldonado-García J. L. García-Mena L. H. Mendieta-Cabrera D. Pérez-Sánchez G. Becerril-Villanueva E. Alvarez-Herrera S. . (2024). Use of extracellular monomeric ubiquitin as a therapeutic option for major depressive disorder. Pharmaceuticals (Basel) 17:841. doi: 10.3390/ph17070841, 39065692 PMC11279398

[ref16] Mellerård J. Edström M. Vrethem M. Ernerudh J. Dahle C. (2010). Natalizumab treatment in multiple sclerosis: marked decline of chemokines and cytokines in cerebrospinal fluid. Multiple Scler. 16, 208–217. doi: 10.1177/1352458509355068, 20007431

[ref17] Mor A. Tankiewicz-Kwedlo A. Krupa A. Pawlak D. (2021). Role of kynurenine pathway in oxidative stress during neurodegenerative disorders. Cells 10:1603. doi: 10.3390/cells10071603, 34206739 PMC8306609

[ref18] Pavón L. Mendieta-Cabrera D. Pérez-Sánchez G. Becerril-Villanueva L. E. Alvarez-Herrera S. Vallejo-Castillo L. . (2024). “Study of neuroendocrine-immune interactions in major depression to develop more efficient therapeutic options,” in PsychoNeuroImmunology Volume 2: Interdisciplinary Approaches to Diseases, (Cham: Springer Nature Switzerland), 147–178.

[ref19] Pérez-Sánchez G. Aguilar-Gómez L. A. Vela-Sancho G. B. Jacinto-Gutiérrez S. Becerril-Villanueva E. Alvarez-Herrera S. . (2026). Reduced peripheral serotonin levels in women with multiple sclerosis: associations with underweight status, treatment duration, and use of interferon beta 1a. Front. Cell. Neurosci. 20:1752975. doi: 10.3389/fncel.2026.1752975, 41890212 PMC13013054

[ref20] Rashid W. Ciccarelli O. Leary S. M. Arun T. Doshi A. Evangelou N. . (2025). Using disease-modifying treatments in multiple sclerosis: Association of British Neurologists (ABN) 2024 guidance. Pract. Neurol. 25, 18–24. doi: 10.1136/pn-2024-004228, 39532459

[ref21] Ricci C. Pivetta M. Martinis E. Valeri V. Colliva C. Giacchè N. . (2025). Tryptophan pathway profiling in multiple sclerosis patients treated with ocrelizumab. Front. Immunol. 16:1603663. doi: 10.3389/fimmu.2025.1603663, 40552280 PMC12183176

[ref22] Siavoshi F. Ladakis D. C. Muller A. Nourbakhsh B. Bhargava P. (2024). Ocrelizumab alters the circulating metabolome in people with relapsing-remitting multiple sclerosis. Ann. Clin. Transl. Neurol. 11, 2485–2498. doi: 10.1002/acn3.52167, 39185939 PMC11537130

[ref23] Skorobogatov K. De Picker L. Verkerk R. Coppens V. Leboyer M. Müller N. . (2021). Brain versus blood: a systematic review on the concordance between peripheral and central kynurenine pathway measures in psychiatric disorders. Front. Immunol. 12:716980. doi: 10.3389/fimmu.2021.716980, 34630391 PMC8495160

[ref24] Stone T. W. Clanchy F. I. L. Huang Y.-S. Chiang N.-Y. Darlington L. G. Williams R. O. (2022). An integrated cytokine and kynurenine network as the basis of neuroimmune communication. Front. Neurosci. 16:1002004. doi: 10.3389/fnins.2022.1002004, 36507331 PMC9729788

[ref25] Stone T. W. Williams R. O. (2023). Modulation of T cells by tryptophan metabolites in the kynurenine pathway. Trends Pharmacol. Sci. 44, 442–456. doi: 10.1016/J.TIPS.2023.04.006 37248103

[ref26] Susin-Calle S. Munteis E. Villoslada P. Martinez-Rodriguez J. E. (2025). The present and future of monoclonal antibody therapies for multiple sclerosis. BioDrugs 39, 815–826. doi: 10.1007/s40259-025-00741-1, 40986178

[ref27] Tauil C. B. da Rocha Lima A. D. Ferrari B. B. da Silva V. A. G. Moraes A. S. da Silva F. M. . (2020). Depression and anxiety in patients with multiple sclerosis treated with interferon-beta or fingolimod: role of indoleamine 2,3-dioxygenase and pro-inflammatory cytokines. Brain Behav Immun Health 9:100162. doi: 10.1016/j.bbih.2020.100162, 34589900 PMC8474597

[ref28] Venkatesan D. Iyer M. Narayanasamy A. Siva K. Vellingiri B. (2020). Kynurenine pathway in Parkinson’s disease—an update. eNeurologicalSci 21:100270. doi: 10.1016/J.ENSCI.2020.100270, 33134567 PMC7585940

[ref29] Wang Y. Zhang Y. Wang W. Zhang Y. Dong X. Liu Y. (2025). Diverse physiological roles of kynurenine pathway metabolites: updated implications for health and disease. Meta 15:210. doi: 10.3390/METABO15030210, 40137174 PMC11943880

[ref30] Zádor F. Joca S. Nagy-Grócz G. Dvorácskó S. Szűcs E. Tömböly C. . (2021). Pro-inflammatory cytokines: potential links between the endocannabinoid system and the kynurenine pathway in depression. Int. J. Mol. Sci. 22:5903. doi: 10.3390/IJMS22115903 34072767 PMC8199129

